# Prediction of potential suitable distribution for sweet cherry (*Prunus avium*) based on the MaxEnt model

**DOI:** 10.1371/journal.pone.0294098

**Published:** 2024-07-24

**Authors:** Hongqun Li, Xiaolong Peng, Peng Jiang, Ligang Xing, Xieping Sun

**Affiliations:** 1 School of Modern Agriculture and Bioengineering, Yangtze Normal University, Chongqing, China; 2 Yan’an Huanglongshan Forestry Bureau, Yan’an, China; Sakarya Uygulamali Bilimler Universitesi, TURKEY

## Abstract

The sweet cherry (*Prunus avium*) is among deciduous fruit trees with high economic value and its planting area is gradually expanding. However, little was known about its accurately suitable area in China. Herein, the potential distributions were modeled based on the MaxEnt model under the current conditions. Its performance was excellent, with AUCs >0.9 for model training and testing. The key environmental factors were the thermal factors (minimum temperature of the coldest month (bio06) from -14.5 to 4.5°C, the mean temperature of the warmest quarter (bio10) from 21.0 to 28.0°C), followed by the water factor (the annual precipitation (bio12) from 500 to 1200 mm), indicating that it is not resistant to cold and heat, nor is it resistant to drought or floods. The suitable area in China mainly is found in seven geographical regions including southwest China (eastern Sichuan, northeast and main urban areas of Chongqing, mid-western Guizhou and mid-northern Yunnan), northwest China (mid-southern Shaanxi, southern Ningxia mid-southern and eastern Gansu), northeast China (Coastal region of Liaoning), central China (most of Henan, mid-northern Hubei and central Hunan), north China (Beijing, Tianjing, mid-southern Shanxi), east China (Shanghai, Jiangsu, Shandong, central Zhejiang, central and northern Anhui and eastern Jiangxi) and south China (western Guangxi). Based on statistical analysis, these fourteen provinces or cities, namely, Shaanxi, Beijing, Tianjing, Shanxi, Hebei, Henan, Shanghai, Jiangsu, Shandong, Sichuan, Guizhou, Yunnan, Liaoning and Hubei were the main regions for current development and utilization while for the twelve provinces with higher moderate suitable areas, namely, Chongqing, Guizhou, Yunnan, Shaanxi, Ningxia, Liaoning, Hubei, Hunan, Zhejiang, Anhui, Jiangxi and Guangxi, we should supplement the appropriate irrigation and winter insulation facilities etc. Additionally, Hubei, Hunan, Anhui, also have been identified to have some potentially suitable areas. These information will help avoid the loss of human labor, material, and financial resources and provide a scientific basis for its current introduction, cultivation, and management.

## Introduction

The sweet cherry (*Prunus avium* Linn), belonging to the family Rosaceae, is one of the earliest mature deciduous fruit trees in northern China and its fruit is rich in nutrients with excellent color, shape, and taste. Hence it attracts domestic and international attention from consumers and is called as the “first branch of spring fruit” and the “treasure” among fruits [1, 2]. Furthermore, it has high medicinal value for humans owing to its abundance of bioactive substances, such as vitamin C and polyphenols [3]. Sweet cherry(Big cherry), originating from southeastern Europe and West Asia, was introduced to Shandong province of China in 1871 and subsequently became an economically beneficial tree species showing a vigorous development trend. For instance, in 2016 its total planting area in China, which ranks first in the world in terms of its cultivation, exceeded 0.18 million square hectometers and its annual output is about 0.7 million ton [4, 5]. Currently, there are three advantageous cultivation areas for sweet cherries in China: the Bohai Bay Rim area, the area along the Longhai Railway and the high-altitude areas in southwestern China [5–7]. For example, some planting areas are found in Shandong (Yantai and Taian), Liaoning (Dalian), Beijing, Hebei (Qinhuangdao), Henan (Zhengzhou), Shaanxi (Xi’an), and Gansu (Tianshui) [5]. Since China’s reform and opening up, sweet cherries have received widespread attention in the cultivation industry owing to their high yield and nutritional value. At present, improvements in living standards with the development of the global economy will inevitably lead to an increasing demand for sweet cherries and its cultivation industry plays a huge role in promoting industrial structure adjustment and increasing farmers’ incomes and wealth [8]. However, some cherry growers may experience economic losses due to improper selection of varieties, rootstocks and suitable cultivation zoning, etc resulting in flowering without bearing fruit for three-to-four years [1, 6]. To satisfy the increasing demand for this fruit in China, it is necessary to expand its growing regions [6, 8]. However, to avoid investment risks mainly evoked by blindly expanding its introduction and cultivation, it is crucial for governments at all levels to conduct research on the national planting division of sweet cherries, propose an index system for its planting division adapted to the climate characteristics of China, and form opinions on zoning for sweet cherries in China.

Fortunately, with the comprehensive application of statistical tools and geographic information systems (GIS), domestic and foreign scholars have widely adopted various models to predict the spatial distribution of target species habitats. In recent years, many species distribution models(SDMs) have become important tools to simulate and visualize the spatial distribution of species and determine key environmental factors that limit a species’ geographical distribution [9, 10]. Specifically, based on a specific algorithm, these SDMs can assess the ecological niche of species by using the target population geographical location, which may obtained from field investigation, specimen records and literatures, and environmental variables affecting their distribution, and then project those niches onto the environment, reflecting the probability of a species’ habitat preferences [11, 12]. However, among the various SDMs (i.e., Bioclim, Domain, MaxEnt and Garp), the MaxEnt model (maximum entropy model) has been confirmed to outperform other SDMs in prediction accuracy, even with incomplete species occurrence data [13–17]. Recently, MaxEnt model has been widely applied in aspects of habitat simulations for target species, the filtering of key environment factors, and quantitative description of environmental factors in target species habitats [15–17]. The advantages of this MaxEnt model selected are as follows: (1) This model only needs presence-only occurrence points (no absence data are needed) and environmental variables for the whole study area; (2)This model may use continuous and categorical variables, and can overcome the correlations to avoid interactions between variables; (3)The computer configuration requirements are relatively low, and it has a user-friendly operation interface; (4) Many studies have also confirmed that this model always has stable and reliable prediction results, even with incomplete data and/or small sample sizes, and the probability distribution generated from this model has a concise mathematical definition amenable to analysis; (5)This model can evaluate the importance of individual environmental variables by using a built-in jackknife test and automatically generate the receiver operating characteristic curves for prediction result verification [9, 14]. Consequently, this MaxEnt model has been extensively adopted for the protection of precious endangered species, management of invasive species, determination of the suitable distributions of target species and prediction of plant diseases and insects, etc. since it was utilized for species distribution prediction in 2004 and already shown unique advantages [9, 17].

For the sweet cherry, its planting area has gradually expanded to the second suitable planting area in southern China, i.e., the middle and lower reaches of the Yangtze River region [18]. However, little was known about its accurately suitable growing area in southern China, which frequently resulted in its blind expansion and economic losses [1, 6]. Therefore, to expand accurately the suitable growing region of sweet cherry, avoid the investment risk of blind expansion, save manpower investigation costs and compensate some equipment to overcome unfavorable conditions, we modeled its potential distribution using this model mentioned above under current conditions based on the known coordinates and related environmental layers selected on their responsibility for species distribution. The aims of this study were: (1) to determine the potential growing areas of the species under the current conditions and (2) to identify the key variables responsible for the potential distributions, which will be helpful for the government at all levels to carry out regional planning of this species in China.

## Materials and methods

### Species distribution samples

The main precise occurrence records from species specimens of *P*. *avium* were acquired from the following four free databases: (1) the Chinese Virtual Herbarium (http://www.cvh.org.cn), (2) the National Specimen Information Infrastructure (http://www.nsii.org.cn/2017/home.php), (3) the Plant Photo Bank of China (http://ppbc.iplant.cn/), (4) the Flora Reipublicae Popularis Sinicae (http://frps.iplant.cn/), (5)other occurrence records for this species that were mainly collected from published scientific articles [5, 7, 18, 19] and our team’s field investigations in Chongqing using a GPS receiver. Owing to the fact that cherries are a cultivated species, some existing points of normal bearing-fruit every year were retained, otherwise they were deleted. If the cherry specimens were clearly incomplete or from a greenhouse, these data were not retained. To avoid errors highly correlated with obvious misidentifications, some data that could not provide accurate geographical locations were deleted, and some duplicate point data regarded as error sources owing to high spatial autocorrelation from the adjacent location [20] were also removed. Consequently, one occurrence point in each grid cell was retained. Finally, 422 occurrence points were retained, and the geographic coordinates of the occurrence points were obtained based on the Gaode Pick Coordinate System (https://lbs.amap.com/tools/picker) or Baidu Pick Coordinate System (http://api.map.baidu.com/lbsapi/getpoint/index.html). To ensure compatibility with the software package MaxEnt, the coordinates of each point were kept in csv format according to the species name, longitude, and latitude, in that order.

### Environmental data

Environmental variables, including temperature, precipitation, and topography etc, can determine the geographical distribution of species [14, 21]. First, we selected 19 bioclimatic variables with 30-arc-second (ca. 1 km^2^ at ground level) spatial resolution under current (i.e., in the period 1970–2000) condition downloaded from the global Worldclim website (http://www.worldclim.org). These bioclimatic variables are equal to the average values from the years 1970–2000, reflecting a combination of annual changes, seasonal characteristics, and extreme environmental conditions [22, 23]. Moreover, altitude (digital elevation model, DEM) data with the above-mentioned spatial resolution were downloaded from the WorldClim website (http://www.worldclim.org) and were adopted to produce the slope and aspect data in ArcGIS 10.2. Secondly, an administrative boundary map of China (1:400 million scale) was acquired from the National Fundamental Geographic Information System (http://nfgis.nsdi.gov.cn/). Finally, these 22 environmental variables, including 19 bioclimatic and 3 terrain variables, were extracted by the boundary maps of China from the above-mentioned global raster data to ensure that all the research areas have the same geographic bounds and cell size.

### Variable selection

Because of the correlation between various environmental factors, if directly applied to the model, there may be an overfitting phenomenon, which is thought to be an error source [20, 24]. To eliminate these negative effects on model building and establish a model that has better performance with fewer variables, these 22 environmental variables were extracted from the corresponding layers associated with 422 documented occurrence points in ArcGIS 10.2, and cross-correlation analysis (Pearson correlation coefficient, *r*) was performed. The decision to include or exclude one of each set of highly correlated variables was made based on the relative predictive power [14, 22]. According to the Pearson correlation coefficient (/r/ ≥ 0.8) in SPSS 21.0 and taking into consideration their relative predictive power (i.e., the importance of each environmental variable to predictor contributions from analysis of predicted results), only one variable with a higher percent contribution from each set of highly cross-correlated variables (/r/≥0.8) was kept in further analyses while the other variable was excluded because of its lower predictive power. Otherwise, all the environmental factors were retained. Finally, 11 remaining variables were retained and utilized for model building (Table 1). Meanwhile, to meet the needs of this MaxEnt model, all remaining variables were converted to asc formats.

**Table 1:** The environmental factors used in this study, their contribution, and permutation importance under current environmental conditions.

Code	Description	Percent contribution	Permutation importance	Code	Description	Percent contribution	Permutation importance
bio06	**Min temperature of coldest month**	**50.9**	**56.3**	Bio14	Precipitation of driest month	2.5	3.4
bio12	**Annual precipitation**	**22.9**	**12.5**	bio03	Isothermality	0.8	1.7
bio10	Mean temperature of warmest quarter	**9.5**	**13.4**	Alt	alt30s1	0.4	2.2
Slo	slope	5.5	4.4	Asp	aspect	0.2	0.2
bio15	Precipitation seasonality	4.3	2.1	bio02	Mean diurnal range	0.1	1.0
bio19	Precipitation of driest month	**2.7**	2.9				

Note: The highlighted variables, selected based on their contributions and permutation importance, are the three main influencing factors.

### Modeling procedure

The potential geographical distributions of *P*. *avium* were modeled by using the MaxEnt model (Version 3.4.1) downloaded freely for scientific research from the world-wide web at http://www.cs.princeton.edu/∼schapire/maxent/, which has been widely utilized to quantify the impact of climate and other environmental changes on a species’ geographical distributions and performed favorably [9, 14]. In the process of model building, 422 known occurrence data of *P*. *avium* and 11 environmental variables were directly imported into the corresponding module of the Maxent model, wherein the training data were 75% of all the occurrence data selected at random, and the remaining 25% of the test data were selected [13, 14]. Meanwhile, a jackknife test and response curve modules were checked in the model interface to calculate habitat suitability curves and estimate the importance of each environmental variable affecting species distribution [22]. The maximum number of background points was set to 10,000. The other settings were the same as described by Li *et al*. (2021) [25]. To guarantee the reliability of the prediction results, the model was run with 10 replicates by cross-validation, and the average habitat suitability was regarded as the final result in a logistic format and asc types [27, 27]. Finally, this result was transformed into a raster format, and the cell value of the prediction results ranged from 0, representing the lowest habitat quality for that species, to 1, representing the highest habitat quality. The Maximum Youden Index (maximum training sensitivity plus specificity logistic threshold) and TPT equilibrium threshold (balance training omission, predicted area, and threshold value) are usually used as cutoff points, providing advantages over other threshold values [28, 29]. And, based on the above cutoff points, the continuous habitat areas for *P*. *avium* were divided into three categories: suitable, moderately, and unsuitable areas.

### Model performance and influencing factors

**Fig 1:**
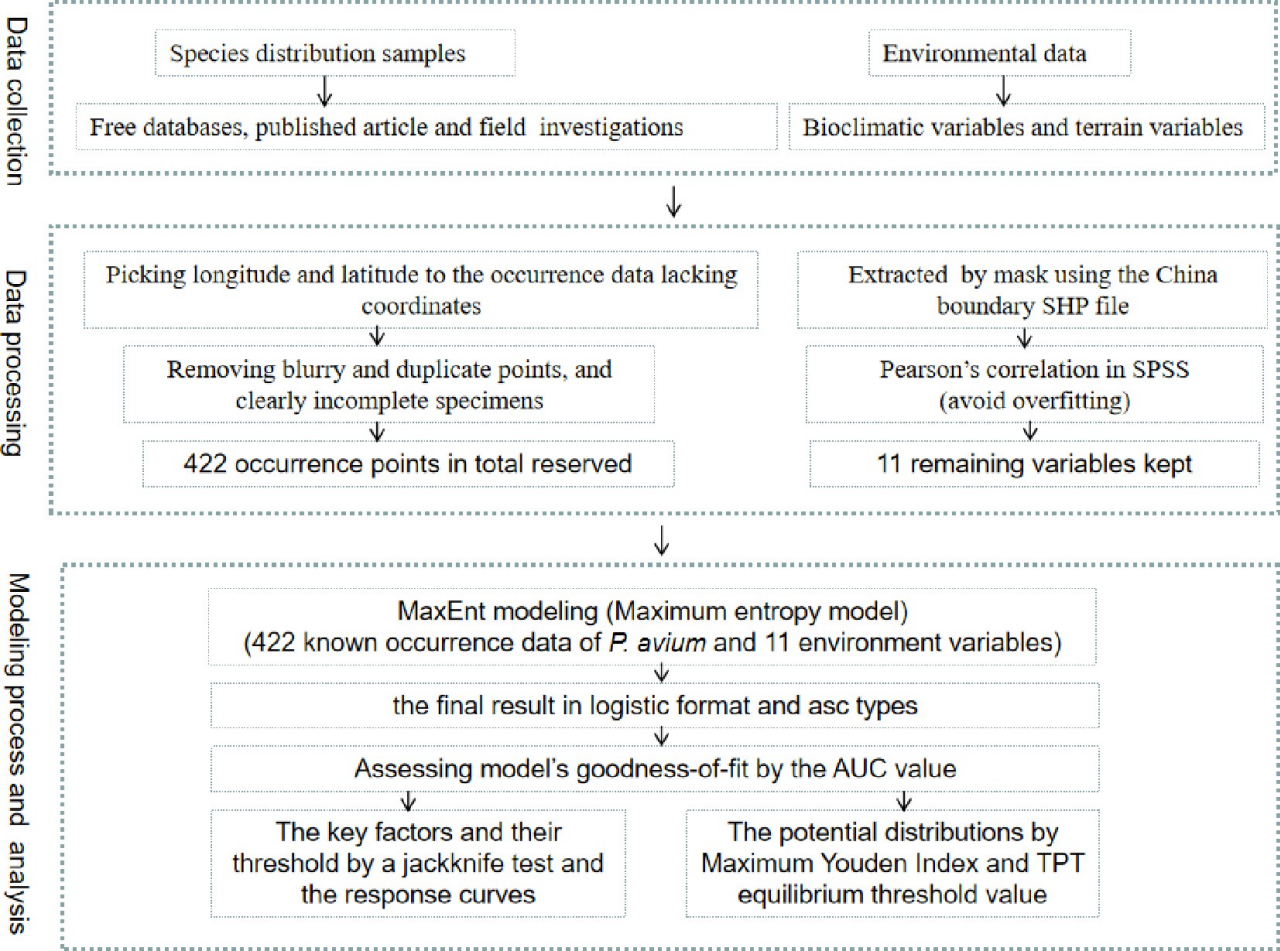
The research flowchart shows a summary of processing methodology, which served as the basis of the analyses.

The area under the receiver operating characteristic curve (AUC) is typically used to assess a model’s goodness-of-fit. Currently, the AUC value is widely regarded as an excellent index for evaluating model performance [14, 30]. The software package MaxEnt (Version 3.4.1) used in our study takes advantage of the AUC value to assess its performance. The AUC value varied from 0.5, implying that the prediction performance was not better than that of the random model or the lowest predictive ability, to 1.0, indicating the best performance or the highest predictive ability [23, 29]. Model performance may be classified as excellent (0.9–1), good (0.8–0.9) or ordinary (0.7–0.8), respectively [31]. According to our regulations, models buliding of this species with values above 0.8 were regarded as useful in our study, whereas AUC values less than 0.8 were not considered in subsequent research. In general, the larger the AUC value, the better the model performance is [31]. By using the software’s built-in jackknife test, based on predictor contributions, permutation importance, and regularized training gain, we can assess the relative influence of individual predictors on the species’ habitat suitability [30, 32, 33]. Furthermore, the response curve generated automatically by the Maxent model was used to show the quantitative relationships between the environmental variables and the logistic probability of occurrence [25, 34]. A flowchart of the study is presented in Fig 1.

## Results

### Modeling evaluation

In this study, the geographic distribution map was generated using the MaxEnt model based on 422 known occurrences of *P*. *avium* and 11 selected environmental variables. Results showed that the AUC values of modeling and testing are close to 1.0 (0.9449 ± 0.00103 for training and 0.9301 ± 0.01197 for testing) under the current conditions (Table 2), suggesting that MaxEnt model performed excellently in predicting the geographic distribution area for the *P*. *avium*.

**Table 2:** The AUC, maximum Youden index and TPT equilibrium threshold generated in 10 replicates.

No.	AUC of model building	AUC of model testing	Maximum Youden index	TPT equilibrium threshold
1	0.9456	0.9264	0.2483	0.0427
2	0.9465	0.9124	0.2317	0.0274
3	0.9443	0.9434	0.2181	0.0402
4	0.9441	0.944	0.182	0.0477
5	0.9441	0.9361	0.2115	0.0499
6	0.9435	0.9429	0.1892	0.0554
7	0.9458	0.9168	0.2374	0.0502
8	0.9461	0.9182	0.2126	0.0536
9	0.9441	0.9365	0.1755	0.0377
10	0.9449	0.9244	0.1901	0.0427
Mean±SD	0.9449± 0.00103	0.9301± 0.01197	0.2096±0.02487	0.0447±0.00841

Note: Maximum Youden Index (maximum training sensitivity plus specificity logistic threshold) and TPT equilibrium threshold (balance training omission, predicted area, and threshold value) in the analysis of the predicted results. AUC(the area under the receiver operating characteristic curve). SD(standard deviation).

### Dominant environmental factors restricting the distribution of the *P*. *avium* in China

**Fig 2:**
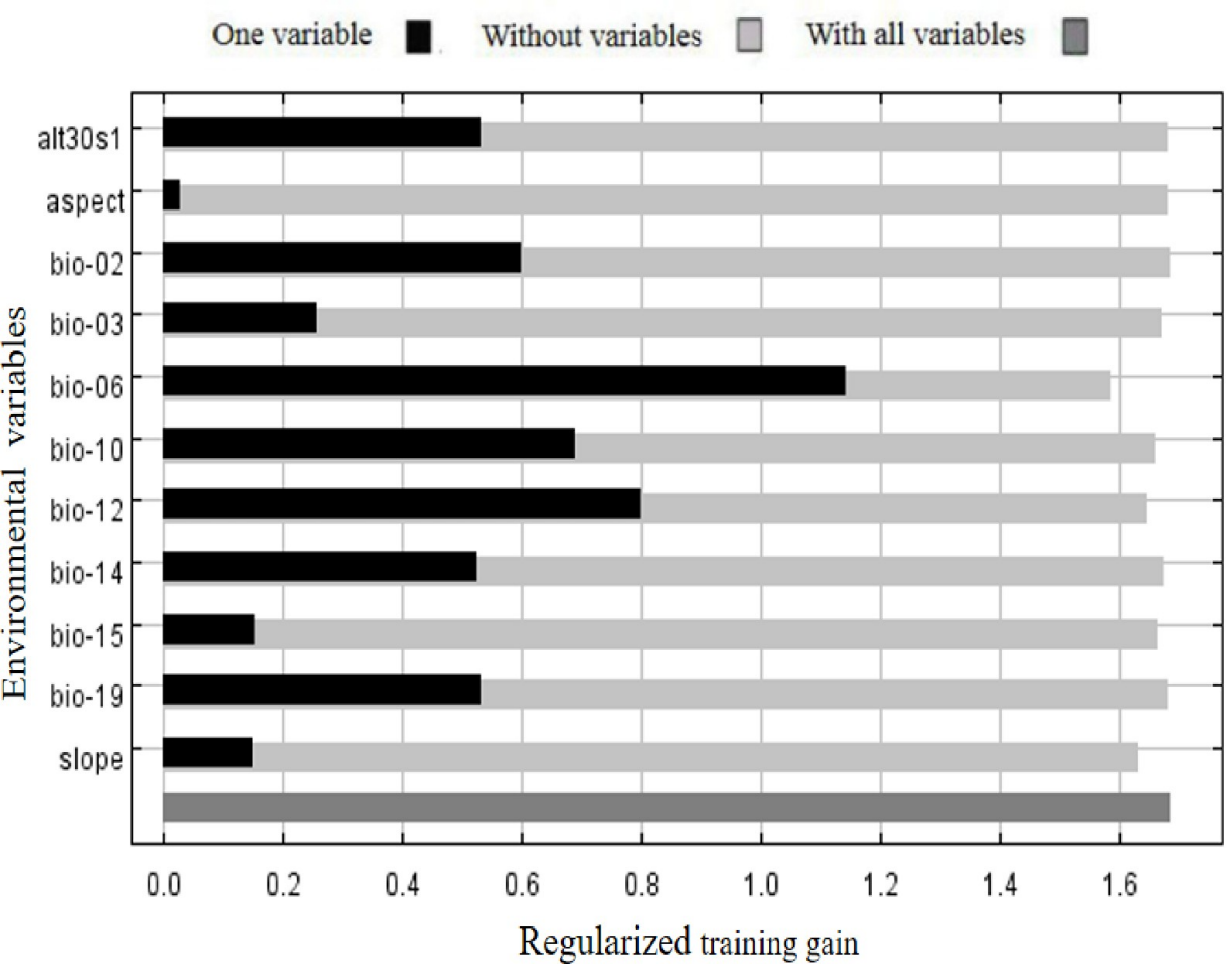
Jackknife test for assessing the relative importance of different environmental variables v to the geospatial distribution of *P*. *avium* under the current condition.

Among the 11 environmental variables, the minimum temperature of the coldest month (bio06), annual precipitation (bio12), and mean temperature of the warmest quarter (bio10) were the strongest predictors associated with the potential distribution of *P*. *avium* relative to the other variables, according to the percent contribution (Table 1). The minimum temperature of the coldest month (bio06) made the largest contribution (50.9%), indicating that this variable significantly affected the distribution of *P*. *avium* under the current conditions, followed by annual precipitation (bio12) and the mean temperature of the warmest quarter (bio10), with 22.9% and 9.5%, respectively. The cumulative contributions of these three factors reached values as high as 83.3%. Meanwhile, based on permutation importance (Table 1), the minimum temperature of the coldest month (bio06) with 56.3% had the highest score (Table 1), followed by annual precipitation (bio12), and the mean temperature of the warmest quarter (bio10) with 12.5% and 13.4%, respectively. The cumulative permutation importance of these three parameters is 82.2%. In addition, based on the built-in jackknife test of the software (Fig 2), the minimum temperature of the coldest month (bio06), annual precipitation (bio12), and the mean temperature of the warmest quarter (bio10) had the highest predictive power (highest regularized training gain) for the geospatial distribution of the *P*. *avium*. Taken together, these three dominant environmental variables identified were minimum temperature of the coldest month (bio06), annual precipitation (bio12), and mean temperature of the warmest quarter (bio10).

### Environmental characteristics of the *P*. *avium* in China

**Fig 3:**
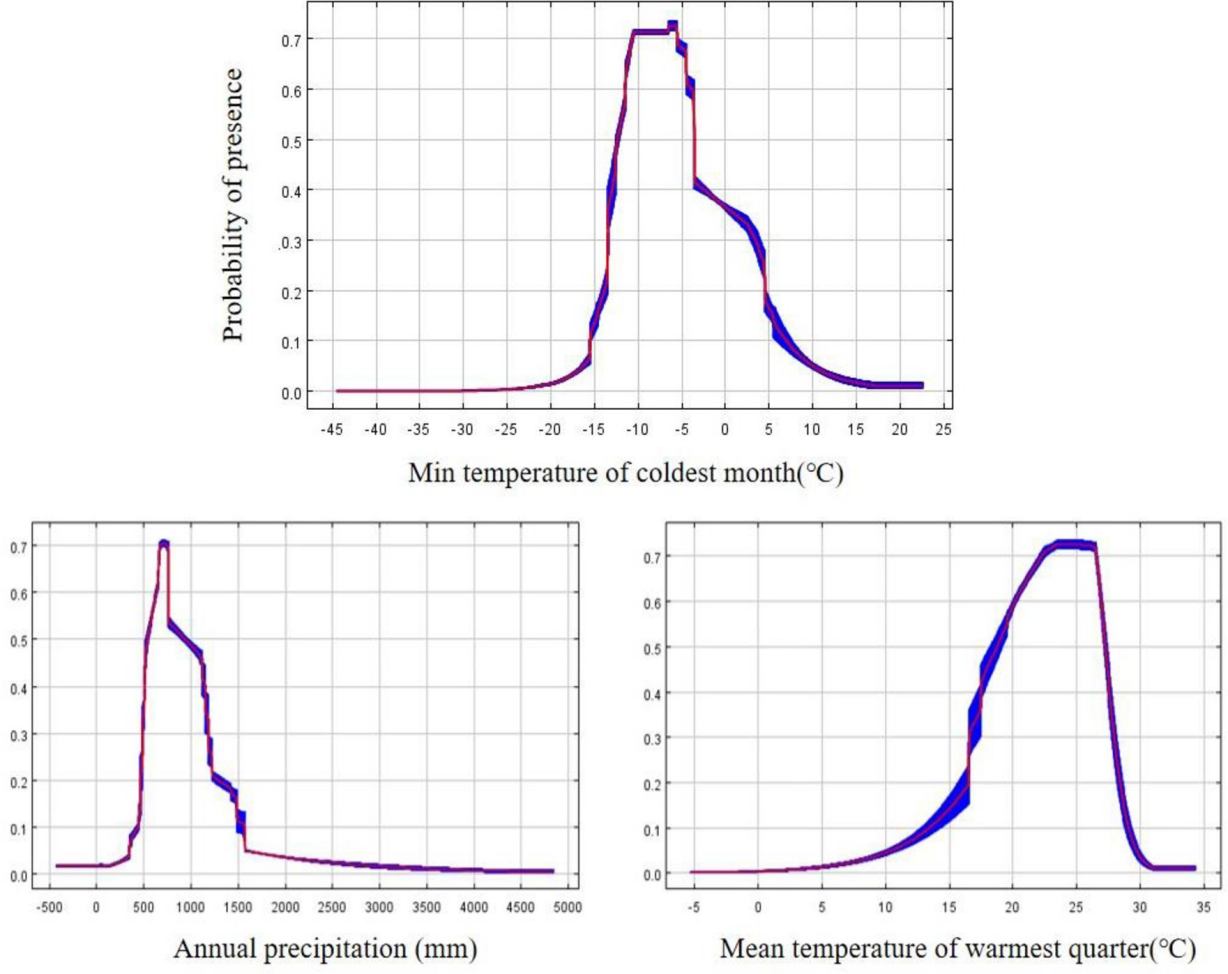
Response curves of MaxEnt-jackknife method and main environment variables.

**Fig 4:**
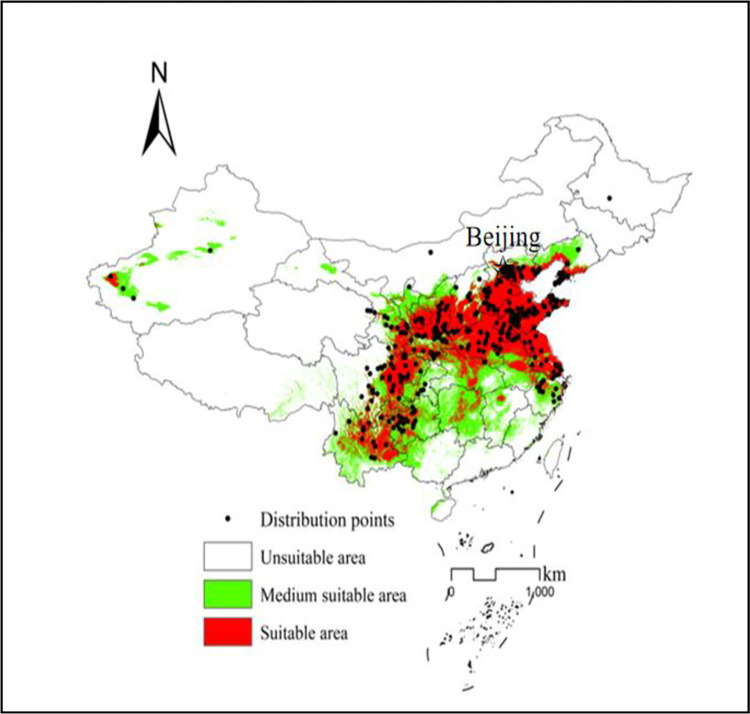
The potential geospatial distribution of *P*. *avium* in China during the periods of 1970–2000.

The area with a fitness level >0.2096 (Maximum Youden Index) was the simulated high-fitness area of *P*. *avium*, which closely matched the actual distribution area (the documented occurrence of *P*. *avium*) (Fig 4), implying that the above-mentioned threshold value is applicable. Therefore, in this study, the threshold of environmental factors was used to show the characteristics of the distribution area of this species in China when the probability of its distribution was >0.2096. Furthermore, to clarify the environmental characteristics of the distribution of this species and obviate interrelated interference among environmental factors, the above three key factors were individually imported into the MaxEnt model. Finally, the individual response curves showed the quantitative relationship between each environmental factor and the logistic probability of presence. As shown in Fig 3, the minimum temperature of the coldest month (bio06) ranges from -14.5 to 4.5°C, the highest point is -6.5°C; the annual precipitation (bio12) ranges from 500 to 1200 mm; and the mean temperature of the warmest quarter (bio10) ranges from 21.0 to 28.0°C.

### Predicting potential distribution

Based on the Maximum Youden Index (average value of 0.2096 in 10 replicates) and TPT equilibrium threshold value (average value of 0.0447 in 10 replicates) as a threshold value under the current condition (Table 1), the final distribution map was reclassified into three categories: unsuitable area (<0.0477), moderately suitable area (0.0447–0.2096), and suitable area (>0.2096). The suitable regions for *P*. *avium* in the study area are mainly concentrated on seven areas including southwest China (eastern Sichuan, northeast and main urban areas of Chongqing, mid-western Guizhou and mid-northern Yunnan), northwest China (mid-southern Shaanxi, southern Ningxia mid-southern and eastern Gansu), northeast China (Coastal region of Liaoning), central China (most of Henan, mid-northern Hubei and central Hunan), north China (Beijing, Tianjing, mid-southern Shanxi), east China (Shanghai, Jiangsu, Shandong, central Zhejiang, central and northern Anhui, and eastern Jiangxi) and south China (western Guangxi). The moderately suitable area lies in southwest China (Chongqing City, Guizhou, eastern Guizhou, western and southern Yunnan and sporadic areas in eastern Sichuan), northwest China (northern Shaanxi, mid-southern Gansu and Ningxia), northeast China (mid-western of Liaoning), central China (sporadic areas in Henan, western and eastern Hubei and most of Hunan), north China (mid-northern Shanxi and eastern Hebei), east China (most of Zhejiang, central and southern Anhui, and most of Jiangxi except for the eastern region), and south China (western Guangxi and northwest Hainan) (Fig 4). Except for the above regions, the other regions in the study area were not suitable for the growth of *P*. *avium*. This region includes most of China, including Tibet, western Ningxia, Qinghai, Xinjiang, Inner Mongolia, Heilongjiang, Jilin, Guangdong, Hainan, and Taiwan. According to the statistical analysis after projection conversion (Fig 4) (Asia_North_Albers_Equal_Area_Conic), the percentages of suitable, moderate, and unsuitable growing regions for *P*. *avium* in China were 6.81%, 13.51%, and 79.68%, respectively. However, for various regions in China (Table 3), to improve the accuracy of projections under the current conditions, the potential suitable areas of *P*. *avium* were calculated based on different projection coordinate systems (https://blog.csdn.net/weixin_42160645/). For example, the projection in the Chongqing area is based on the WGS 1984 UTM ZONE 48N because its longitude range is 102°–108° (Table 3). The percentages of suitable areas in 14 provinces or cities, including Shaanxi, Beijing, Tianjing, Shanxi, Hebei, Henan, Shanghai, Jiangsu, Shandong, Sichuan, Guizhou, Yunnan, Liaoning and Hubei, reached by 21.70% to 92.47%, indicating that these 14 provinces or cities are the main regions of current development and utilization, The moderately suitable areas mainly exist in Chongqing, Guizhou, Yunnan, Shaanxi, Ningxia, Liaoning, Hubei, Hunan, Zhejiang, Anhui, Jiangxi and Guangxi based on the percentage of the moderate predicted area from 24.22% to 66.51%. To expand accurately the moderately suitable growing region of the sweet cherry, the appropriate irrigation and winter insulation facilities etc. should be supplemented in these provinces. Moreover, compared to their actual distributions, Hubei, Hunan, Anhui, etc also have potentially suitable areas.

**Table 3:** Commonly used projection coordinate systems in China.

Belt Number	Longitude range (East longitude)	Central meridian longitude	Data sources
43N	72°–78°	75°	https://blog.csdn.net/
44N	78°–84°	81°	
45N	84°–90°	87°	
46N	90°–96°	93°	
47N	96°–102°	99°	
48N	102°–108°	105°	
49N	108°–114°	111°	
50N	114°–120°	117°	
51N	120°–126°	123°	
52N	126°–132°	129°	
53N	132°–138°	135°	

## Discussion

Sweet cherries are rich in bioactive substances, such as vitamin C and polyphenols, and are known as “vitamin pills” by experts [6, 35]. This indicates high medicinal value too [3]. If the human body lacks these elements, corresponding diseases occur, such as iron deficiency anemia, vitamin A deficiency-related dry skin and scurvy. They are healthy fruits too that prolong people’s lives, especially with significant beauty effects, so they are very popular among women and present a strong sales momentum. However, owing to their intolerance towards long-term storage and transportation in the past, their cultivation ranges have long been very narrow, resulting in slow expansion and limited yields, which cannot meet market needs. Fortunately, recently with the improvement of cultivation technology and the rapid development of the modern storage and transportation industry, their cultivation range and yields in China are continuously expanding, and the demand for sweet cherries has gradually increased with the improvement of living standards. However, little is known about the potentially suitable areas for sweet cherries, especially in the southern regions, which results in investment risks mainly evoked by blindly expanding its introduction and growing area. Fortunately, with the development of applied ecology, many SDMs have been widely adopted to identity the potential distributions of many species based on presence and absence data and in practice, it is very difficult to obtain absence data [31]. Among many SDMs, the predictive ability of the MaxEnt model with presence-only occurrence data is always stable and reliable and it outperforms other SDMs [16]. In this study, its potential distributions were modeled by using the Maxent model under current conditions based on only 422 precise coordinates of species occurrences (in practice, it is very difficult to obtain absent data) together with 11 environmental layers, and its predictive performance was excellent according to the evaluation results of AUC index. Furthermore, based on the above-mentioned prediction outcomes (Fig 4), the suitable area matched closely with the actual distribution of sweet cherries, indicating that their predicted results were precise and the above threshold value under the current condition was reasonable and reliable. There are three advantageous cultivation areas for sweet cherries in China. One is the Bohai Bay Rim area, which is dominated by Shandong (Yantai and Taian), Liaoning (Dalian), Beijing, and Hebei (Qinhuangdao), the other is the area along the Longhai Railway, dominated by Henan (Zhengzhou), Shaanxi (Xi’an), and Gansu (Tianshui) [5] and the third one is the newly added high-altitude areas in northwest and southwest China [7]. These results are similar to our predicted results based on this model. However, the model also identified some new potentially suitable areas for sweet cherries in China, which indicates that sweet cherries have not yet reached their full potential range (Fig 4). For example, Hubei, Hunan, Anhui, also have been identified to have some potentially suitable areas. As shown in Fig 4 and Table 4, in southwest China, the percentages of the suitable growing areas in Sichuan, Guizhou, and Yunnan provinces respectively exceed 20%, while it only is 10.99% in Chongqing City. In northwest China, those in Gansu and Ningxia provinces are <20% while it reaches 63.01% in Shaanxi. In northeast China, it is only 21.70% in Liaoning. In north China, those in Beijing, Tianjing, Shanxi, and Hebei exceed 20%. In central China, those in Henan and Hubei provinces exceed 20% while it reaches 11.01% in Hunan. In east China, those in Shanghai, Jiangsu, and Shandong exceed 20%, while those in Zhejiang, Anhui, and Jiangxi are <20%; In south China, those in Hainan and Guangxi are <20%. Through comprehensive analysis, the percentages of the suitable growing areas in Sichuan, Guizhou, Yunnan, Shaanxi, Liaoning, Beijing, Tianjing, Shanxi, Hebei, Hubei, Henan, Shanghai, Jiangsu and Shandong exceeded by 21.70% to 92.47% (Table 4), indicating that these fourteen provinces or cities are the main regions for their current development and utilization. In addition, the percentages of moderately suitable-growing areas in Chongqing, Guizhou, Yunnan, Shaanxi, Ningxia, Liaoning, Hubei, Hunan, Zhejiang, Anhui, Jiangxi and Guangxi provinces or cities ranged from 24.22% to 62.07% (Table 3), indicating that these twelve provinces or cities may introduce some special varieties or appropriate irrigation or winter insulation facilities etc for local development with deliberation.

**Table 4:** Area and percentage of habitat distribution for *P*. *avium* in different provinces or cities in 1970–2000.

Region^a^	Various regions in China	Predicted area/km^2^			Percentage of predicted area/%		
		Suitable habitat	Moderately suitable habitat	Unsuitable habitat	Suitable habitat	Moderately suitable habitat	Unsuitable habitat
Chongqing	Southwest China	9513.65	45177.6	31874.2	10.99	52.19	36.82
Sichuan		151734	88011.2	278628	29.27	16.98	53.75
Guizhou		49994.5	107669	22831.9	27.70	59.65	12.65
Yunnan		119587	157353	129234	29.44	38.74	31.82
Shaanxi	Northwest China	139484	64364.7	17505	63.01	29.08	7.91
Gansu		76680.1	66958.2	304846	17.10	14.93	67.97
Ningxia		8427.96	38163.8	14893.6	13.71	62.07	24.22
Liaoning	Northeast China	32661	51277.7	66553	21.70	34.07	44.22
Beijing	North China	13645.9	2033.07	2234.04	76.18	11.35	12.47
Tianjing		11692.4	0	0	100	0	0
Shanxi		81039.3	21164.2	63227.3	48.99	12.79	38.22
Hebei		109848	15832.6	69953.2	56.15	8.09	35.75
Henan	Central China	150270	23831.3	951.687	85.84	13.61	0.54
Hubei		51682.6	89752.6	51631	26.77	46.49	26.74
Hunan		24135.3	145736	49261.9	11.01	66.51	22.48
Shanghai	East China	6190.69	0	0	100	0	0
Jiangsu		99373.3	8094.7	0.7751	92.47	7.53	0.00
Zhejiang		18421.7	47428.9	38364.7	17.68	45.51	36.81
Anhui		18642.8	54059.4	71485	12.93	37.49	49.58
Jiangxi		4180.82	54493.7	114440	2.42	31.48	66.11
Shandong		167904	319.895	0	99.81	0.19	0
Hainan	South China	0	4781.32	30729.2	0	13.46	86.55
Guangxi		6127.69	59994.3	181559	2.47	24.22	73.30

^a^ Except for Chongqing, Beijing, Tianjing, Shanghai, and Tianjing as cities, all others as provinces.

The potential distribution area of a species is strongly correlated with environmental variables including temperature, rainfall, and terrain [24, 27]. Among the environmental variables, climate (temperature and rainfall) is one of the most critical factors limiting the potential distribution of species [27]. Based on the predictor contributions, permutation importance, and regularized training gain, the dominant environmental variables identified were temperature (the minimum temperature of the coldest month [bio06] and the mean temperature of the warmest quarter [bio10]) and rainfall (the annual precipitation [bio12]), wherein the minimum temperature of the coldest month (bio06) made the greatest contributions to the distribution model for *P*. *avium*, indicating that thermal factors are the dominant climate factors affecting the distribution of sweet cherry in China, followed by water factors during the growing season. This conclusion is supported by previous studies [19, 36]. Specifically, it was reported that the critical low temperature during the winter dormancy period of sweet cherry should not be lower than -20°C, which is considered the critical temperature for winter damage in large cherries [19, 36, 37]. On the contrary, if it falls below this critical temperature, it is difficult for the sweet cherries to safely overwinter. For example, its main trunk and branches are prone to frost cracking and gummosis, while flower buds are also susceptible to frost damage. For example, at the critical temperature range of -21–-24°C, the entire cherry tree dies from an increased electrolyte leakage rate, which limits the northward development of sweet cherries [36]. For the Yunnan and Guizhou provinces, the main factor restricting the cultivation of sweet cherry is insufficiently cold temperatures in winter. Consequently, the fruit tree often blooms but does not bear fruit [7]. If the temperature is too high in summer, cherry trees exhibit excessive vigor, branches grow too fast, canopy closure is increased, and fruit quality is poor, resulting in a loss of commercial value. Most seriously, high temperatures in summer can cause abnormal differentiation of flower buds in the early stages to produce a large number of deformed flowers, such as the twin-room flower, which forms a “twin fruit” in the coming year [38–40]. Furthermore, we have shown that the sweet cherry can live in the minimum temperature of the coldest month (bio06), which ranges from -14.5 to 4.5°C,the highest presence is at -6.5°C, which ensures maximum flowering and fruiting. Meanwhile, the mean temperature of the warmest quarter (bio10) ranges from 21.0 to 28.0°C. In addition, sweet cherries can be easily grown in areas with an annual precipitation of 600–800 mm, according to past studies [6, 7, 18]. This was highly consistent with our research results, namely, the annual precipitation (bio12) ranged from 500 to 1200 mm in China.

In summary, based on 422 selected known occurrence data and 11 environment variables identified, the distribution of sweet cherries has been simulated using the MaxEnt Model. The predicted suitable area of the sweet cherries matched closely with its actual distribution, suggesting that the predicted results are reasonable and correct and can guide our practical activities from this optimization methods. Based on the three dominant environmental variables identified from 11 environment variables, the sweet cherries is not resistant to cold and heat, and nor is it resistant to drought or flooding, indicating that it needs strict survival conditions. However, it is difficult to change the three dominant variables influencing its geographical distribution, including the minimum temperature of the coldest month (bio06), the mean temperature of the warmest quarter (bio10), and the annual precipitation (bio12). To avoid investment risks, we should select these fourteen provinces or cities, namely Shaanxi, Beijing, Tianjing, Shanxi, Hebei, Henan, Shanghai, Jiangsu, Shandong, Sichuan, Guizhou, Yunnan, Liaoning and Hubei as key areas for planting and promotion, while for the ten provinces with higher moderate suitable areas, we should supplement the appropriate irrigation and winter insulation facilities or introduce some special varieties etc. In addition, other environmental variables, such as soil, solar radiation, wind speed, extreme weather, disease, and human deforestation, were not considered in this study. These variables may have non-negligible effects on the habitat distribution of sweet cherries. Therefore, additional environmental factors should be considered in the future to improve the prediction accuracy of the MaxEnt model.

## Conclusions

1. The suitable area of the *P*. *avium* in China mostly is located in southwest China (eastern Sichuan, the northeast and main urban areas of Chongqing, mid-western Guizhou, and mid-northern Yunnan), northwest China (mid-southern Shaanxi, southern Ningxia, mid-southern and eastern Gansu), northeast China (the coastal region of Liaoning), central China (most of Henan, mid-northern Hubei, and central Hunan), north China (Beijing, Tianjing, mid-southern Shanxi), east China (Shanghai, Jiangsu, Shandong, central Zhejiang, central and northern Anhui, and eastern Jiangxi) and south China (western Guangxi). (2) Fourteen provinces or cities (Shaanxi, Beijing, Tianjing, Shanxi, Hebei, Henan, Shanghai, Jiangsu, Shandong, Sichuan, Guizhou, Yunnan, Liaoning and Hubei) were the main regions for their current development and utilization, while for the twelve provinces with higher moderate suitable areas, we may supplement the appropriate irrigation and winter insulation facilities etc. Additionally, Hubei, Hunan, Anhui, also have been identified to have some potentially suitable areas.(3)The key factors selected were: the minimum temperature of the coldest month (bio06) ranged from -14.5 to 4.5°C, with the highest presence is -6.5°C; annual precipitation (bio12) ranging from 500 to 1200 mm; the mean temperature of the warmest quarter (bio10) ranging from 21.0 to 28.0°C. During the growing season, the thermal factors were the dominant climatic factors affecting the distribution of sweet cherries in China, followed by water factors, indicating that it is not resistant to cold and heat, nor is it resistant to drought or floods.
